# Surgical outcomes and patient selection in nonagenarians with colon cancer: a comparative multi-institutional study of laparoscopic and open approaches

**DOI:** 10.1007/s00423-025-03911-5

**Published:** 2025-11-27

**Authors:** Ryohei Shoji, Fuminori Teraishi, Satoe Takanaga, Toshiharu Mitsuhashi, Ryo Inada, Toshiaki Toshima, Tsuyoshi Ohtani, Ryosuke Yoshida, Naoto Hori, Kaoru Shigemitsu, Sumiharu Yamamoto, Tetsushi Kubota, Yuka Okano, Tetsuji Nobuhisa, Fumitaka Taniguchi, Wataru Ishikawa, Tatsuo Matsuda, Tatsuo Umeoka, Toshiyoshi Fujiwara, Setouchi Colorectal Neoplasm Registration study group collaborators

**Affiliations:** 1 Department of Gastroenterological Surgery, Okayama University Graduate School of Medicine, Dentistry and Pharmaceutical Sciences, Okayama, 700-8558 Japan; 2 Department of Surgery, NHO Fukuyama Medical Center, Hiroshima, Japan; 3 Center for Innovative Clinical Medicine, Okayama University Hospital, Okayama, Japan; 4 Department of Surgery, Kochi Health Sciences Center, Kochi, Japan; 5 Department of Surgery, Kagawa Rosai Hospital, Marugame, Japan; 6 Department of Surgery, Saiseikai Okayama Hospital, Okayama, Japan; 7 Department of Surgery, Okayama Rosai Hospital, Okayama, Japan; 8 Department of Surgery, Tottori Municipal Hospital, Tottori, Japan; 9 Department of Surgery, Tsuyama Chuo Hospital, Tsuyama, Japan; 10 Department of Surgery, Okayama City Hospital, Okayama, Japan; 11 Department of Surgery, Kobe Red Cross Hospital, Kobe, Japan; 12 Department of Surgery, Onomichi City Hospital, Onomichi, Japan; 13 Department of Surgery, Himeji Red Cross Hospital, Himeji, Japan; 14 Department of Surgery, National Hospital Organization Iwakuni Clinical Center, Iwakuni, Japan; 15 Department of Surgery, Fukuyama City Hospital, Fukuyama, Japan; 16 Department of Surgery, Matsuda Hospital, Kurashiki, Japan; 17 Department of Surgery, Matsuyama City Hospital, Matsuyama, Japan

**Keywords:** Oldest-old patients, Colon cancer, Laparoscopic surgery, Surgical outcome, Overall survival

## Abstract

**Purpose:**

The appropriate surgical approach for colon cancer (CC) in nonagenarian patients remains a subject of clinical debate. This study aimed to compare the short-term outcomes of laparoscopic (Lap) versus open (Open) surgery in patients aged ≥ 90 years with resectable colon cancer.

**Methods:**

This multi-institutional retrospective cohort study included oldest-old patientswith pathological Stage II/III CC who underwent elective surgery at 15 hospitals between 2011 and 2022. Patients with rectal cancer, Stage 0/I/IV disease, or emergency surgery were excluded. To address selection bias, inverse-probability-weighted regression adjustment and stabilized inverse probability of treatment weighting (sIPTW) were applied. The primary outcome was postoperative complications; secondary outcomes included overall survival (OS).

**Results:**

Median age was 92 years in both groups. Before adjustment, the Lap group had a higher proportion of female patients (*p* = 0.038) and lower ASA scores (*p* = 0.01). Laparoscopic surgery was associated with a significantly longer operative time (220 vs. 171 min, *p* = 0.046) but less intraoperative blood loss (10 vs. 78 mL, *p* < 0.01). Postoperative complication rates were comparable (Lap: 31.8%, Open: 33.8%), while the Lap group had a significantly shorter hospital stay (13 vs. 17 days, *p* < 0.01). D3 lymph node dissection was more frequently performed in the Lap group (*p* < 0.01). After sIPTW, overall survival did not differ significantly between groups (*p* = 0.61).

**Conclusion:**

Both laparoscopic and open surgery are feasible options for selected nonagenarians with colon cancer. Laparoscopic surgery may offer benefits in terms of reduced blood loss and shorter hospitalization, despite longer operative times. Careful patient selection considering frailty and comorbidities is essential in determining the most appropriate surgical approach.

## Introduction

Colon cancer (CC) remains a significant cause of morbidity and mortality worldwide [1]. While advances in screening and treatment have improved prognosis, global aging presents unique challenges in managing CC in the extremely older, particularly those aged 90 years and older. As CC incidence increases with age, this vulnerable population requires an individualized approach to diagnosis and treatment [1, 2]. Although colonoscopy remains the gold standard for CC screening, its indications and safety in the extremely older warrant careful consideration [3, 4].

Surgical resection represents a cornerstone of CC treatment; however, its role in the oldest older remains controversial due to increased risks of postoperative complications and mortality [2]. Several studies have suggested that laparoscopic colorectal resection offers advantages in older patients, including reduced blood loss, shorter hospital stays, and faster recovery [5–9]. Nevertheless, data specifically focusing on outcomes of various surgical approaches, including minimally invasive surgery, in extremely older patients with CC remain limited. While some studies suggest the feasibility and potential benefits of laparoscopic surgery in this age group [6–8, 10], the optimal surgical strategy remains undefined.

Therefore, this multi-institutional retrospective study aims to evaluate outcomes across different surgical approaches in CC resection patients aged 90 years and older, with the goal of informing clinical decision-making regarding the optimal surgical approach.

## Materials and methods

The ethics committee of Okayama University Hospital approved this retrospective study (approval number 2112–036) and all participating hospitals approved this study as exempt human subject research. Study data were collected and managed using Research Electronic Data Capture (REDCap) tools hosted at Okayama University Hospital. We also followed the recommendations of the Equator Network and complied with the Strengthening the Reporting of Observational Studies in Epidemiology (STROBE) Statement: guidelines for reporting observational studies.

### Study population

**Fig. 1:**
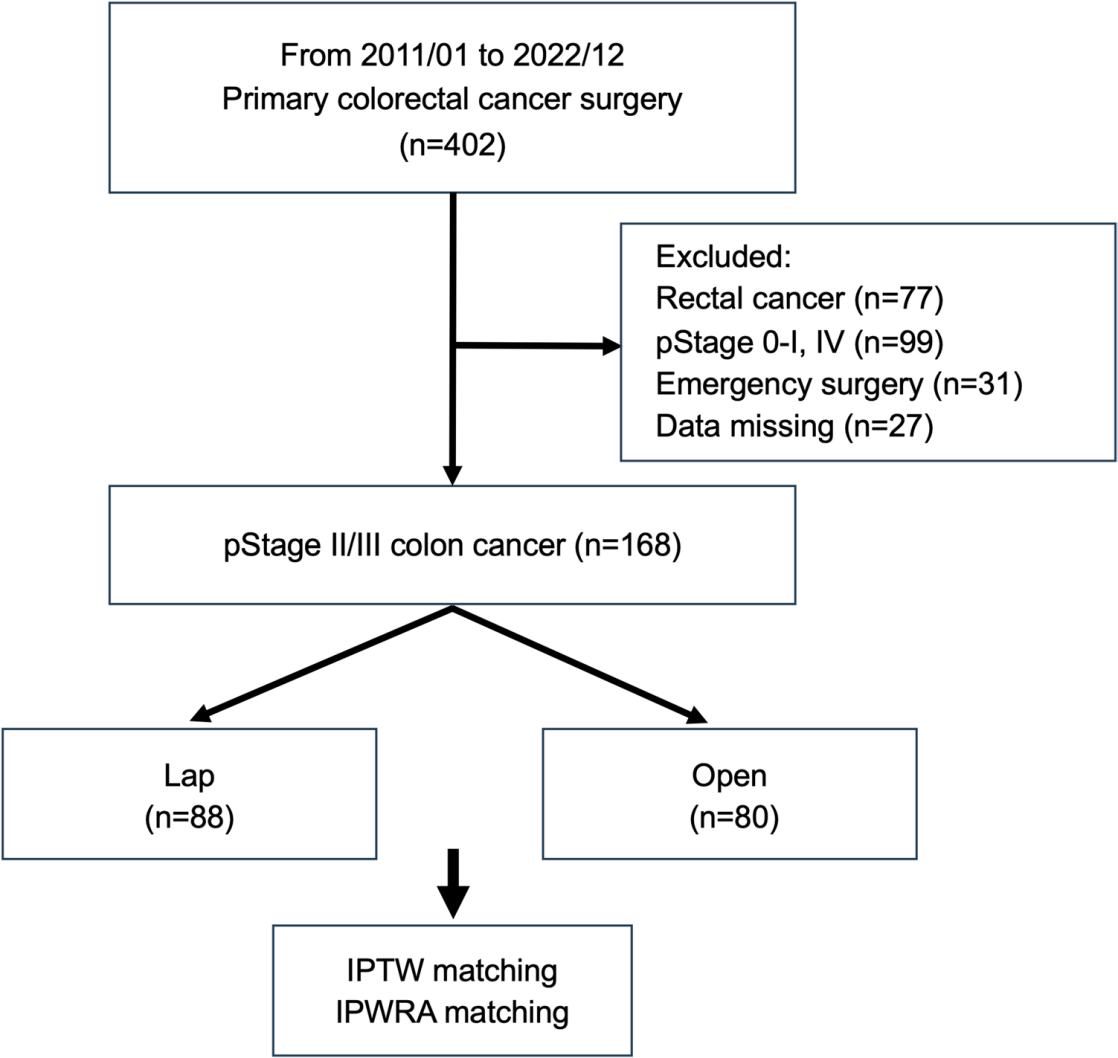
Flow diagram of patient selection process. Abbreviations: *IPWRA* inverse-probability-weighted regression adjustment; *IPTW* inverse probability of treatment weighting; *pStage* pathological Stage

This retrospective, cohort study used data from the Setouchi Colorectal Neoplasm Registration study database, which collected information on oldest-old patients aged 90 years or older from 15 Okayama University-affiliated hospitals between January 2011 and December 2022. A total of 403 cases of colorectal cancer in oldest-old patients were identified. The inclusion criteria were patients with colorectal cancer undergoing laparoscopic surgery or open surgery for pathological Stage II or III cancer, and exclusion criteria included rectal cancer, Stage 0-I cancer, Stage IV cancer, case of emergency surgery, and cases with missing data (Fig. 1).

### Research electronic data capture (REDCap)

REDCap is a secure, web-based software platform designed to support data capture for research studies, providing 1) an intuitive interface for validated data capture; 2) audit trails for tracking data manipulation and export procedures; 3) automated export procedures for seamless data downloads to common statistical packages; and 4) procedures for data integration and interoperability with external sources [11, 12].

### Outcome measurement

Incidence of postoperative complications was evaluated according to the Clavien–Dindo classification [13, 14]. Any deviation from normal postoperative status was considered a complication, and the treatment required to correct the complication was the basis for the grade of severity. Thus, complications that required medical therapy were classified as Grade II; complications that required surgical, endoscopic, or radiologic treatment as Grade III; life-threatening complications as Grade IV; and postoperative death as Grade V. In this study, Grade II or higher were considered as having postoperative complications. Complications that occurred in the first 30 days after surgery were registered by the physician in charge. The primary outcome was the incidence of all postoperative complications according to the Clavien–Dindo classification. Complications were recorded for 30 days after surgery by the physician-in-charge.

### Patient follow-up

In accordance with the guidelines of the Japanese Society of Colorectal Cancer [15], postoperative surveillance, including serum carcinoembryonic antigen levels (every 3 months), computed tomography (CT) (every 6 months), and colonoscopy (every 2 years), was usually performed for 5 years after surgery.

### Statistical analysis

Statistical analysis of continuous variables was expressed as median (interquartile range), and categorical variables were expressed as number (%). Categorical variables were compared between the open surgery (Open) and laparoscopic surgery (Lap) groups using Fisher's exact test or chi-square test, while continuous variables were compared using the Mann–Whitney U test.

For post-operative complications, differences between Open and Lap groups were calculated as risk ratios and their 95% confidence intervals (CIs) using modified Poisson regression. 95% CIs were calculated using the bootstrap method. Inverse-probability-weighted regression adjustment (IPWRA), which is doubly robust, was employed to adjust for confounding factors [16]. In the IPWRA treatment model (i.e., inverse probability of treatment weighting (IPTW)), propensity scores were calculated using a logistic regression model, with age, gender, tumor location, ASA classification, pathological stage, and lymph node dissection as explanatory variables and surgical approach (Lap or Open) as the response variable. These propensity scores were used to calculate the stabilized IPTW (sIPTW) in the IPWRA method using the following formula:

In Lap group,

$$weight=\frac{Pr(laparoscopic \;surgery)}{Propemsity \;Score}$$

In Open group,

$$weight=\frac{Pr(open \;surgery)}{1-Propemsity \;Score}$$

In the IPWRA outcome model (i.e., regression adjustment), a modified Poisson regression was performed with the same variables to calculate the propensity score and estimate the risk of post-operative complications.

For survival time analysis to identify prognostic factors, the Cox proportional hazards model was employed to calculate hazard ratios and their 95% confidential interval. The sIPTW method was performed to adjust for confounding factors. In the sIPTW analysis for the Cox model, propensity scores were estimated, and weights were computed for adjustment, with postoperative complications added as covariates in addition to those used in the IPWRA model. Overall survival (OS) was calculated using the Kaplan–Meier method, and the two groups were compared using the log-rank test.

All statistical analyses were conducted using Stata/MP (version 18.0, Stata Corp, College Station, TX, USA) and GraphPad Prism 6 (GraphPad Software, Boston, MA, USA). A *p* < 0.05 was considered statistically significant. Because this study has an exploratory component, a multiplicity of tests was not considered.

## Results

### Patient characteristics and clinical features

The study analyzed 168 patients, comprising 88 in the laparoscopic surgery group (Lap group) and 80 in the open surgery group (Open group). Patient characteristics, surgical outcomes, and pathological findings are presented in Table 1. The median age was 92 years in both cohorts. The Lap group had a significantly higher proportion of female patients compared to the Open group (73.9% versus 58.7%, *p* = 0.038). Regarding the American Society of Anesthesiologists (ASA) classification, the Open group demonstrated a significantly higher proportion of ASA class 3 patients (72.5% versus 50%, *p* = 0.01). Right-sided colonic tumors predominated in both groups (Open: 64 cases; Lap: 60 cases). D3 lymph node dissection was performed more frequently in the Lap group (77.3% versus 50%, *p* < 0.01).

**Table 1:** Baseline patient characteristics, surgical outcome, and pathological outcome

	All	Open	Lap	*p* value
	*n* = 168	*n* = 80	*n* = 88	
Age, yrs, median (IQR)	92 (91–93)	92 (91–93)	92 (91–94)	0.79
Gender				**0.038**
Male	56 (33.3)	33 (41.3)	23 (26.1)	
Female	112 (66.7)	47 (58.7)	65 (73.9)	
BMI, kg/m2, median (IQR)	20.2 (18.1–22.0)	20.0 (18.1–21.6)	20.5 (18.1–22.9)	0.14
History of abdominal surgery, *n* (%)	45 (26.8)	21 (26.3)	24 (27.3)	0.88
ASA classification				**0.01**
2	64 (38.1)	22 (27.5)	42 (47.8)	
3	102 (60.7)	58 (72.5)	44 (50)	
4	1 (0.6)	0	1 (1.1)	
unknown	1 (0.6)	0	1 (1.1)	
Tumor location				0.08
Right	124 (73.8)	64 (80)	60 (68.2)	
Left	44 (26.2)	16 (20)	28 (31.8)	
Lymph node dissection				** < 0.01**
D3	108 (64.3)	40 (50)	68 (77.3)	
non-D3	58 (34.5)	40 (50)	18 (20.5)	
unknown	2 (1.2)	0	2 (2.2)	
Surgical outcome				
Operation time, min, median (IQR)	195 (142–244)	171 (123–210)	220 (161–258)	**0.046**
Blood loss, ml, median (IQR)	30 (10–100)	78 (10–144)	10 (5–50)	** < 0.01**
Blood transfusion, *n* (%)	12 (7.1)	8 (10)	4 (4.5)	0.17
Post operative complications				
C-D Grade I-V, n (%)	55 (32.7)	27 (33.8)	28 (31.8)	0.79
C-D Grade III-V, n (%)	36 (21.4)	17 (21.3)	19 (21.6)	0.96
30-day mortality, n (%)	0 (0)	0 (0)	0 (0)	−
Length of stay, days, median (IQR)	14 (11–20)	17 (13–24)	13 (11–18)	** < 0.01**
Reoperation, *n* (%)	1 (0.6)	1 (1.3)	0 (0)	0.34
Pathological outcome				
Tumor size, mm, median (IQR)	55 (40–70)	60 (44–72)	45 (40–66)	0.06
Harvested lymph node, median (IQR)	17 (11–25)	16 (8–25)	18 (13–25)	0.09
Proximal margin, mm, median (IQR)	80 (60–120)	80 (55–120)	90 (63–120)	0.67
Distal margin, mm, median (IQR)	90 (60–110)	80 (50–109)	100 (70–110)	**0.046**
Lymphartic invasion, *n* (%)	101 (60.1)	48 (60)	53 (60.2)	0.98
Vascular invasion, *n* (%)	117 (69.6)	58 (72.5)	59 (67)	0.44
pStage, *n* (%)				0.87
II	104 (61.9)	49 (61.3)	55 (62.5)	
III	64 (38.1)	31 (38.7)	33 (37.5)	

Abbreviations: *ASA* American Society of Anesthesiologists, *BMI* body mass index, *C − D* Clavien-Dindo classification, *IQR* interquartile range, *pStage* pathological Stage

Surgical outcomes analysis revealed longer operative times in the Lap group (220 versus 171 min, *p* = 0.046) but significantly reduced intraoperative blood loss (10 versus 78 ml, *p *< 0.01). The overall postoperative complication rates were comparable between groups (Open: 33.8%; Lap: 31.8%). Similarly, the incidence of Clavien-Dindo grade II or higher complications showed no significant difference (Open: 21.3%; Lap: 21.6%). No 30-day mortality occurred in either group. The Lap group demonstrated significantly shorter postoperative hospital stays (13 versus 17 days, *p* < 0.01). One patient in the Open group required reoperation due to wound dehiscence.

Pathological examination showed comparable tumor sizes and harvested lymph node counts between groups. The Lap group achieved significantly longer distal resection margins (100 versus 80 mm, *p* = 0.046). Pathological staging distribution was similar between groups, with Stage II disease present in 49 Open group and 55 Lap group patients, and Stage III disease in 31 Open group and 33 Lap group patients.

### IPWRA and sIPTW analysis

The study employed inverse probability weighted regression adjustment (IPWRA) using six variables (age, gender, tumor location, ASA classification, pathological stage, and lymph node dissection) for postoperative complications analysis. sIPTW incorporated these factors plus postoperative complications for overall survival analysis. Following sIPTW, previously observed significant differences in clinical characteristics were eliminated, achieving improved balance between groups (Table 2).

**Table 2:** Patient characteristics after inverse probability of treatment weighting (IPTW) and inverse probability weighted regression adjustment (IPWRA)

	Unweighted				After sIPTV for OS				After sIPTW for postoperative complication			
	Open	Lap			Open	Lap			Open	Lap		
*N*	80	88	SMD	VR	79	86	SMD	VR	79	86	SMD	VR
Variables used in propensity score calculation												
Age, yrs, median [IQR]	92.00 [91.00, 93.00]	92.00 [91.00, 93.50]	0.04	1.42	92.00 [91.00, 93.00]	92.00 [91.00, 93.00]	0.02	1.24	92.00 [91.00, 93.00]	92.00 [91.00, 93.00]	0.03	1.23
Gender, *n*(%)												
Female	47 (58.8%)	65 (73.9%)	−0.33	0.78	52.56 (66.8%)	58.64 (67.9%)	−0.02	0.98	52.79 (67.0%)	58.40 (67.6%)	−0.01	0.99
Male	33 (41.2%)	23 (26.1%)			26.09 (33.2%)	27.72 (32.1%)			26.06 (33.0%)	27.99 (32.4%)		
Tumor location, *n*(%)												
Left	16 (20.0%)	28 (31.8%)	−0.24	1.32	18.09 (23.0%)	20.87 (24.2%)	−0.03	1.03	18.23 (23.1%)	20.79 (24.1%)	−0.02	1.03
Right	64 (80.0%)	60 (68.2%)			60.56 (77.0%)	65.49 (75.8%)			60.61 (76.9%)	65.60 (75.9%)		
ASA classification, *n*(%)												
ASA 2	22 (27.5%)	42 (47.7%)	−0.42	1.25	29.99 (38.1%)	33.74 (39.1%)	−0.02	1.01	30.15 (38.2%)	33.76 (39.1%)	−0.02	1.01
ASA 3/4	58 (72.5%)	46 (52.3%)			48.66 (61.9%)	52.63 (60.9%)			48.69 (61.8%)	52.63 (60.9%)		
pStage, *n*(%)												
II	49 (61.3%)	55 (62.5%)	−0.03	0.98	47.63 (60.6%)	50.48 (58.4%)	0.04	1.02	47.91 (60.8%)	50.36 (58.3%)	0.05	1.02
III	31 (38.8%)	33 (37.5%)			31.01 (39.4%)	35.89 (41.6%)			30.94 (39.2%)	36.02 (41.7%)		
Postoperative complication, *n*(%)												
No	53 (66.2%)	60 (68.2%)	−0.05	0.96	54.32 (69.1%)	58.27 (67.5%)	0.03	1.03				
Yes	27 (33.8%)	28 (31.8%)			24.32 (30.9%)	28.10 (32.5%)						
Lymphnode dissection, *n*(%)												
D3	40 (50.0%)	68 (79.1%)	−0.63	0.66	51.67 (65.7%)	57.80 (66.9%)	−0.03	0.98	51.90 (65.8%)	57.83 (66.9%)	−0.02	0.98
non-D3	40 (50.0%)	18 (20.9%)			26.98 (34.3%)	28.57 (33.1%)			26.95 (34.2%)	28.56 (33.1%)		
Surgical outcomes (unadjusted due to being an intermediate factor)												
Operation time, min, median [IQR]	170.50 [125.50, 210.00]	220.00 [163.00, 257.50]	0.46	1.10	180.00 [141.00, 210.00]	216.00 [168.00, 257.00]	0.41	1.02	180.00 [141.00, 210.00]	216.00 [171.00, 257.00]	0.43	1.04
Blood loss, ml, median [IQR]	77.50 [10.00, 144.00]	10.00 [5.00, 50.00]	−0.24	2.42	80.00 [10.00, 140.00]	10.00 [5.00, 50.00]	−0.17	2.90	80.00 [10.00, 140.00]	10.00 [5.00, 50.00]	−0.17	2.89
Blood transfusion												
No	72 (90.0%)	83 (95.4%)	−0.21	0.49	71.01 (90.3%)	81.34 (94.8%)	−0.17	0.56	71.38 (90.5%)	81.25 (94.7%)	−0.16	0.58
Yes	8 (10.0%)	4 (4.6%)			7.64 (9.7%)	4.43 (5.2%)			7.47 (9.5%)	4.53 (5.3%)		
Pathological outcome (unadjusted, as it is unknown at the time of surgical procedure selection)												
Tumor size, mm, median [IQR]	60.00 [45.00, 70.00]	50.00 [40.00, 65.00]	−0.23	0.79	58.00 [45.00, 70.00]	50.00 [40.00, 70.00]	−0.14	0.89	58.00 [45.00, 70.00]	50.00 [40.00, 70.00]	−0.14	0.89
Harvested lymph node, median [IQR]	16.00 [8.50, 24.00]	18.00 [13.00, 25.00]	0.30	0.86	18.00 [10.00, 27.00]	18.00 [13.00, 25.00]	0.11	0.86	18.00 [10.00, 27.00]	18.00 [13.00, 25.00]	0.11	0.85
Proximal margin, mm, median [IQR]	80.00 [55.00, 120.00]	90.00 [65.00, 120.00]	−0.14	0.48	80.00 [60.00, 120.00]	90.00 [70.00, 120.00]	−0.14	0.49	80.00 [60.00, 120.00]	90.00 [70.00, 120.00]	−0.14	0.49
Distal margin, mm, median [IQR]	80.00 [50.00, 108.00]	100.00 [70.00, 110.00]	0.27	0.78	80.00 [60.00, 110.00]	100.00 [70.00, 110.00]	0.25	0.77	80.00 [60.00, 110.00]	100.00 [70.00, 110.00]	0.26	0.78
Lymphartic invasion, *n*(%)												
No	32 (40.0%)	35 (39.8%)	0.15	0.90	30.39 (38.6%)	32.52 (37.7%)	0.17	0.88	30.70 (38.9%)	31.98 (37.0%)	0.19	0.86
Yes	48 (60.0%)	53 (60.2%)			48.26 (61.4%)	53.84 (62.3%)			48.15 (61.1%)	54.41 (63.0%)		
Vascular invation, *n*(%)												
No	22 (27.5%)	29 (33.0%)	−0.18	1.27	21.19 (26.9%)	26.71 (30.9%)	−0.15	1.21	21.30 (27.0%)	26.29 (30.4%)	−0.13	1.20
Yes	58 (72.5%)	59 (67.0%)			57.45 (73.1%)	59.65 (69.1%)			57.55 (73.0%)	60.10 (69.6%)		

Abbreviations: *SD* standard deviation, *SMD* standardized mean difference, *VR* variance ratio

### Risk of postoperative complications

After sIPTW, the risk ratio for postoperative complications in the Lap group compared to the Open group was 1.16, which did not reach statistical significance (Table 3).

**Table 3:** Risk ratio of postoperative complications following laparoscopic surgery compared to open surgery

	Risk ratio	95% confidence interval	*p*-value
Unadjusted	0.94	(0.61, 1.45)	0.790
Adjusted by IPWRA	1.16	(0.71, 1.91)	0.544

### Survival analysis

**Fig. 2:**
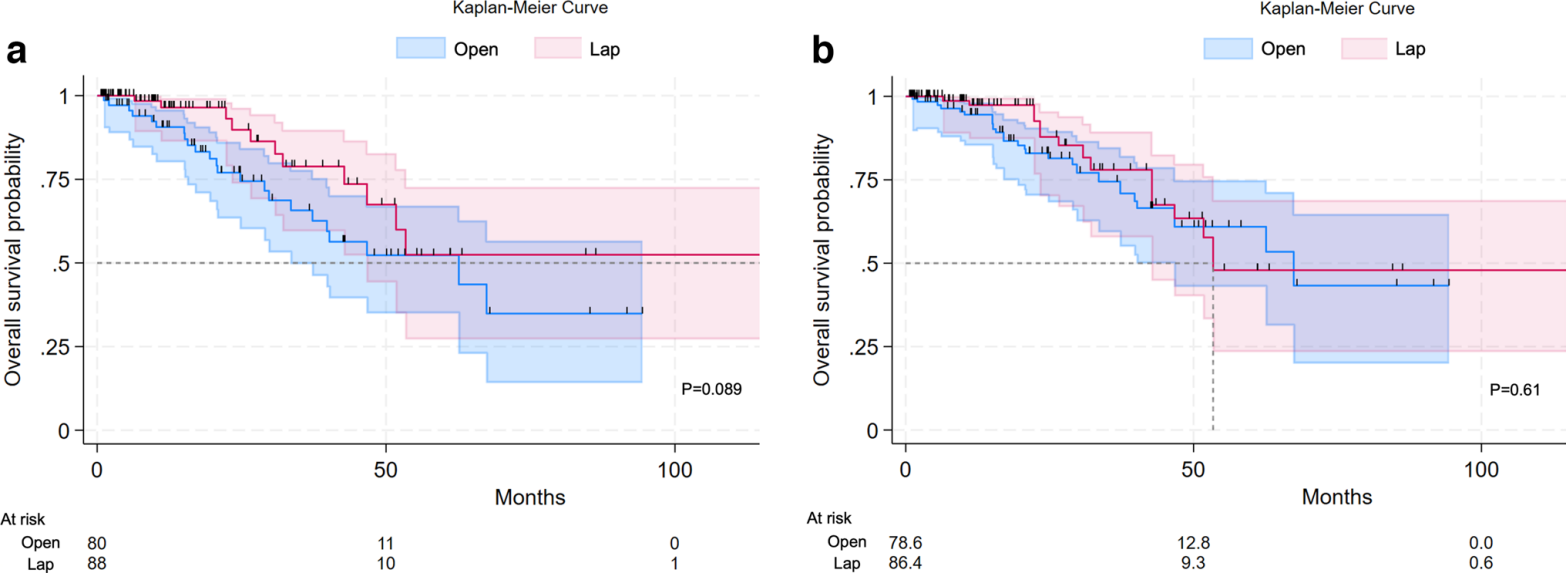
Kaplan–Meier curves depicting overall survival after open versus laparoscopic surgery, adjusted by inverse probability of treatment weighting (IPTW). a) Overall survival prior to IPTW adjustment, b) Overall survival after IPTW adjustment in patients with Stage II/III colon cancer. a) *P* = 0.089 (log-rank test). b) *P* = 0.61 (log-rank test)

Unadjusted Kaplan–Meier survival analysis suggested a trend toward improved survival in the Lap group (*p* = 0.089) (Fig. 2a). However, after sIPTW, survival curves showed no significant difference between groups (*p* = 0.61) (Fig. 2b). Cox proportional hazards regression analysis, using the Open group as reference, yielded hazard ratios of 0.54 (unadjusted) and 0.82 (adjusted) for the Lap group, neither achieving statistical significance (Table 4). Three-year survival rates after sIPTW were comparable between groups (Open: 74.5%; Lap: 78.0%) (Table 5).

**Table 4:** Mortality hazard ratio for overall survival following laparoscopic surgery compared to open surgery (Cox proportional hazard model)

OS	Hazard ratio	95% confidence interval	*p*-value
Unadjusted	0.54	(0.26, 1.11)	0.094
Adjusted by sIPTW	0.82	(0.40, 1.71)	0.605

**Table 5:** Three-year and five-year survival rates derived from survival time analysis

Unadjusted			
	Time	Survival	95% confidence interval
Open	36	0.6572	(0.4980, 0.7766)
	60	0.523	(0.3507, 0.6697)
Lap	36	0.7883	(0.5960, 0.8964)
	60	0.5246	(0.2730, 0.7257)
Adjusted by sIPTW			
	Time	Survival	95% confidence interval
Open	36	0.7446	(0.5944, 0.8460)
	60	0.6092	(0.4307, 0.7470)
Lap	36	0.7799	(0.5789, 0.8931)
	60	0.4791	(0.2353, 0.6878)

## Discussion

The management of colorectal cancer in nonagenarians presents a unique clinical challenge, requiring a nuanced approach that balances oncological principles with the inherent physiological limitations of this age group. This multi-institutional retrospective study aimed to compare the outcomes of laparoscopic and open surgery in patients aged 90 years and older, contributing to the growing body of evidence regarding optimal surgical approaches in this population. Our findings indicate that laparoscopic surgery in this cohort, while associated with longer operative times and a greater proportion of D3 lymph node dissections, offers potential benefits such as reduced intraoperative blood loss and shorter postoperative hospital stays. Notably, a higher proportion of female patients and those with an ASA class of 2 were included in the laparoscopic group, suggesting a potential selection bias towards patients deemed more suitable for a minimally invasive approach. However, after sIPTW, no significant difference was observed in postoperative complications or long-term survival between the two surgical approaches.

Previous studies have indicated that colorectal cancer surgery can be performed safely in carefully selected nonagenarians, with associated favorable long-term survival outcomes, as demonstrated by Roque-Castellano et al. [17]. Hashimoto et al. [18] investigated short- and long-term survival following curative resection for colorectal cancer in patients over 90 years of age, emphasizing the importance of meticulous patient selection and surgical expertise. Jeon et al. [19] compared surgical and non-surgical treatment modalities for colorectal cancer in nonagenarians, suggesting that surgery may contribute to improved survival outcomes in appropriately selected individuals. Similarly, Tamura et al. [20] reported acceptable postoperative outcomes following elective surgery for colorectal cancer in nonagenarians who underwent careful preoperative screening.

The observed benefits of laparoscopy, including reduced blood loss and shorter hospital stays, align with previous literature highlighting the potential advantages of minimally invasive surgery in older adults. Kolarsick et al. [21] emphasized the role of minimally invasive surgery in minimizing the impact of colorectal surgery in older patients, potentially leading to faster recovery and reduced morbidity. Chesney et al. [22] suggested that older adults may derive the greatest benefit from minimally invasive surgery for sigmoid and rectal cancer. A systematic review by Devoto et al. [23] found that laparoscopic resection in very older patients resulted in decreased morbidity, shorter hospital stays, and a trend toward improved survival compared to open surgery. However, it is important to acknowledge that the evidence remains heterogeneous, and a consistent survival benefit has not been universally demonstrated.

Several studies have specifically examined the feasibility and safety of laparoscopic colectomy in the oldest-old. Franco et al. [24] reported findings from the CLIMHET study group, demonstrating the feasibility and safety of laparoscopic right colectomy in oldest-old patients with colon cancer. Similarly, Hashida et al. [25] presented a single-center analysis of laparoscopic surgery for colorectal cancer in super-older patients, suggesting its potential benefits in this challenging population. Wang et al. [26], utilizing propensity score matching, concluded that laparoscopic surgery is a safe and effective approach in older patients with colon cancer, consistent with our findings of reduced morbidity and shorter hospital stays. However, the longer operative times associated with laparoscopy in our study merit careful consideration. Prolonged anesthesia and surgical stress can be particularly detrimental in frail older patients. Therefore, patient selection is paramount. Comprehensive geriatric assessment may serve as a valuable tool for preoperatively evaluating frailty and predicting postoperative outcomes [27]. In our previous study, we demonstrated that multidisciplinary prehabilitation was effective in preventing postoperative complications in frail older patients with colorectal cancer [28]. However, in the present multi-institutional study, there was no standardized perioperative care protocol across the participating institutions, and detailed information regarding postoperative management, including the use of enhanced recovery after surgery programs, was not available. This limitation should be taken into consideration when interpreting the results, as differences in perioperative management may have influenced postoperative outcomes.

The finding that the laparoscopic group had a greater proportion of D3 lymph node dissections raises questions regarding the extent of surgical resection in this population. While D3 lymph node dissection has been shown to improve recurrence rates in older patients with colon cancer [29], the potential benefits must be weighed against the increased risk of complications associated with more extensive surgery. Hwang et al. [30] specifically investigated laparoscopic complete mesocolic excision with D3 lymph node dissection for right colon cancer in older patients, highlighting the feasibility of this approach. In our study, the absence of a significant survival difference despite the higher rate of D3 dissection in the laparoscopic group suggests that the extent of lymph node dissection may not be the primary determinant of long-term outcome in this age group. This observation aligns with the study by Niemeläinen et al. [31], which demonstrated that long-term survival after elective colon cancer surgery in the aged is significantly influenced by factors beyond the surgical technique itself.

Interestingly, we observed a longer distal resection margin in specimens from the laparoscopic group. The clinical significance of this finding remains unclear. While adequate surgical margins are crucial for oncological control, limited evidence suggests that a longer distal margin confers a survival advantage [32]. Further research is needed to determine whether this observation reflects differences in surgical technique or variations in tumor biology.

As with all retrospective studies, this research is subject to inherent limitations. The retrospective design introduces the potential for selection bias and confounding variables. Although IPWRA and sIPTW was employed to mitigate these biases, residual confounding may still be present. Furthermore, while this analysis leverages multi-institutional pooled data, the relatively small sample size limits the statistical power to detect subtle differences in outcomes. The inherent heterogeneity within the nonagenarian population also presents a challenge. Although sIPTW was conducted based on ASA classification in this study, comorbidities, functional status, and cognitive impairment can all significantly influence postoperative outcomes. Future investigations should incorporate more comprehensive assessments of frailty and comorbidity to better stratify patients and tailor surgical approaches. Zeng et al. [33] conducted a multi-center retrospective study in nonagenarians, underscoring the importance of considering individual patient characteristics when making treatment decisions. Another limitation lies in the fact that only overall survival could be analyzed for prognostic assessment. While cancer-specific survival analysis would have been optimal, although we could confirm mortality status, it proved challenging to accurately assess recurrence patterns and specific causes of death. The primary factor contributing to this limitation was the difficulty in conducting follow-up assessments after hospital discharge, largely due to changes in family circumstances and living environments. These challenges in maintaining long-term follow-up significantly impacted our ability to collect comprehensive survival data, particularly regarding disease-specific outcomes.

## Conclusion

Our study suggests that both laparoscopic and open surgery can be safely performed in selected nonagenarian patients with colorectal cancer. Laparoscopic surgery offers potential advantages in terms of reduced blood loss and shorter hospital stays, but may be associated with longer operative times. Patient selection based on frailty, comorbidity, and functional status is paramount. Future research should focus on developing more refined risk stratification tools and optimizing perioperative care to improve outcomes in this challenging patient population. The decision regarding the optimal surgical approach should be individualized, taking into account the patient's overall health, tumor characteristics, and the surgeon's expertise.

## Data Availability

The data generated and analyzed during the present study are not publicly available for privacy and ethical reasons, but are available from the corresponding author upon reasonable request.
